# Internal fixation of anterior acetabular fractures with a limited pararectus approach and the anatomical plates: preliminary results

**DOI:** 10.1186/s12891-021-04034-w

**Published:** 2021-02-18

**Authors:** Sheng Yao, Kaifang Chen, Fengzhao Zhu, Jia Liu, Yulong Wang, Lian Zeng, Yizhou Wan, Yanzhen Qu, Liang Yang, Xiaodong Guo, Xu Yang

**Affiliations:** 1 Department of Orthopaedics, Union Hospital, Tongji Medical College, Huazhong University of Science and Technology, Wuhan, China; 2 Department of Radiology, Union Hospital, Tongji Medical College, Huazhong University of Science and Technology, Wuhan, China; 3 Department of Orthopaedics, Suizhou Hospital, Hubei university of medicine, Suizhou, China

**Keywords:** Minimally invasive, Acetabular fractures, Quadrilateral plate, Anatomical plate, Pararectus approach

## Abstract

**Background:**

The surgical treatment of acetabular fracture has adverse outcomes and high risk, and minimally invasive method is a good way to reduce complications and improve hip joint function. This study is to investigate the treatment of certain acetabular fractures primarily involving the anterior column and quadrilateral plate using a limited pararectus approach and the anatomical plates.

**Methods:**

A consecutive cohort of 17 patients with anterior displaced acetabular fractures were managed operatively with a limited approach and the anatomical plates. Ten patients had anterior column fractures, 1 patient had anterior wall fracture, 4 patients had transverse fractures and 2 patients had anterior column with posterior hemi-transverse fractures. The inferior half of the pararectus approach was adopted to open the medial window and to access the anterior column and the quadrilateral plate. The anatomical plates were used for internal fixation. Residual displacements were assessed on the postoperative CT scans using a standardized digital method. The surgical details, hip functional outcomes, and complications were noted.

**Results:**

All of the patients were operated using the limited pararectus approach and the anatomical plates successfully. The mean operative time and blood loss were 90.9 min and 334.1 ml, respectively. The average postoperative residual gap and step displacement on CT were 2.9 mm and 0.7 mm, respectively. The radiological outcome was estimated according to the Matta score, ten of the cases were graded anatomical, six were graded imperfect, and one was graded poor. Follow up averaged 15 months. Functional outcomes were excellent for nine, good for six, and fair for two. It was noted that one case of peritoneal injury was repaired intraoperatively.

**Conclusions:**

The limited pararectus approach with the advantages of less trauma, direct exposure to the anterior column and quadrilateral plate. The anatomical plates can fit with the surface of the acetabulum, which saves the time of remodeling plates during operation and facilitate fracture reduction. The combination approach can be a good choice for limited surgery of displaced anterior acetabular fractures especially involving the quadrilateral plate.

**Supplementary Information:**

The online version contains supplementary material available at 10.1186/s12891-021-04034-w.

## Background

Multiple surgical approaches and implantations have been invented and modified for the treatment of acetabular fractures [1–6]. In 1960s, Letournel [1] described the standardized surgical treatment strategies, and some other approaches and algorithms have also contributed greatly to the management of acetabular fractures. However, open reduction and internal fixation of acetabular fractures are still accompanied by high hip replacement and dysfunction rate because of the complex anatomy and extensive surgical wound [7–9]. Several studies have shown that limited incision or minimally invasive methods with less tissue injuries and surgical complications can achieve good clinical results in the treatment of partially displaced acetabular fractures [10–12]. Even though, it has not been widely used for acetabular fractures in clinical practice because blind manipulation under insufficient exposure makes anatomical reduction and fixation very difficult.

The pararectus approach had advantages of adequate visualization of the dome and quadrilateral plate, less complications, and easy to achieved anatomical reduction [3, 13–15]. The fully exposed medial window of this approach enables direct visualization of the anterior column and quadrilateral plate, and allows palpation of the anterior aspect of the sacroiliac joint. An oblique mini-incision similar to the medial window of the pararectus approach was used to acquire closed reduction under finger palpate combined with screw fixation for acetabular transverse fractures [10]. The suprapectineal and infrapectineal plates are the most common methods for management of acetabular fractures involving the anterior column and quadrilateral plate [16, 17]. Generally, the remodeling of plate is very difficult due to the limitation of small incision and will increase operation time consumption [11].

This limited pararectus approach uses the medial window of the pararectus approach, bypassing the rectus abdominis and accessing closer to the anterior acetabulum, pelvic brim, and quadrilateral plate. The purpose of the preliminary study was to introduce our clinical experience in the treatment of acetabular fractures using the new approach and the anatomical suprapectineal and infrapectineal plates. We hypothesis that the limited approach could provide enough visualization for reduction and fixation of anterior acetabular fractures, and the anatomical plates, without the need for remodeling intraoperatively, could fit into the irregular acetabulum surface and facilitate the operation.

### Patients and methods

Inclusion criteria:

1. Suffering from closed acetabular fracture with anterior column
2. The operation was performed using the limited pararectus approach and anatomical plate
3. Injury to operation time is less than 2 weeks.

Exclusion criteria:

1. Fractures without displacement
2. Fractures involving iliac wing or posterior wall requiring open reduction
3. Delayed or open acetabular fracture;
4. Age <  18 years

After institutional review board approval, a total of 17 consecutive cases of anterior acetabular fractures at a Level 1 trauma centre were treated operatively from March 2015 to December 2018 (Table 1). There were 12 males and 5 females, with an average age of 46.9 (range: 28–78, SD: 14.1). The fracture patterns were ten for anterior column, one for anterior wall, two for transverse, and four for anterior column with posterior hemitransverse. The initial causes of injury include traffic accident (10, 58.8%), fall from height (≧3 m)(6, 35.3%), and industrial accident (1,5.9%). The average time from injury to surgery was 6.8 days. All displaced acetabular fractures were routinely treated with skeletal traction preoperatively to relieve pain and maintain temporary reduction of hip joint. Open reduction and internal fixation (ORIF) were done through the limited approach, with longitudinal traction of the ipsilateral limbs and lateral traction of the greater trochanter, in needed. Deep venous thrombosis was prevented by low molecular weight heparin (2000 IU, qd). The operative time, intraoperative haemorrhage, perioperative complications and follow-up data were noted.

**Table 1:** Patient demographics and peri-operative data overview

No.	Sex	Age	Classification^a^	Injury to surgery (day)	Operative time (min)	Blood loss (ml)	Internal Fixation	Complications
1	M	48	AC	3	45	100	Supra-pectineal plate	No
2	F	39	AW	4	50	150	Supra-pectineal plate	No
3	M	47	AC	4	80	350	Supra-pectineal plate	No
4	F	40	TR	10	110	550	Infra-pectineal and ilioischiatic plates	No
5	F	35	TR	14	100	750	Infra-pectineal and ilioischiatic plates	Peritoneal injury
6	M	45	AC	3	90	150	Supra- and infra-pectineal plates	No
7	M	71	AC	8	105	400	Supra-pectineal plate	No
8	M	60	TR	8	115	200	Infra-pectineal and ilioischiatic plates	No
9	M	29	AC	5	70	150	Supra- and infra-pectineal plates	No
10	M	28	AC	10	65	300	Infra-pectineal plate	No
11	F	44	TR	7	110	450	Infra-pectineal and ilioischiatic plates	No
12	M	47	AC-PHT	9	105	560	Infra-pectineal and ilioischiatic plates	No
13	M	78	AC	5	85	250	Supra- and infra-pectineal plates	No
14	M	54	AC	7	50	200	Supra- and infra-pectineal plates	No
15	M	60	AC	6	90	350	Supra- / infra-pectineal plates and ioischiatic plates	No
16	F	43	AC-PHT	4	150	450	Supra-pectineal plate	No
17	M	30	AC	9	125	320	Supra- and infra-pectineal plates	No
Mean and standard deviation		46.9 ± 14.1		6.8 ± 3.0	90.9 ± 28.7	334.1 ± 177.3		

^a^ Letournel’s classification, *AC* anterior column, *AW* anterior wall, *PHT* posterior hemi transverse, *TR* transverse

### Surgical technique

**Fig. 1:**
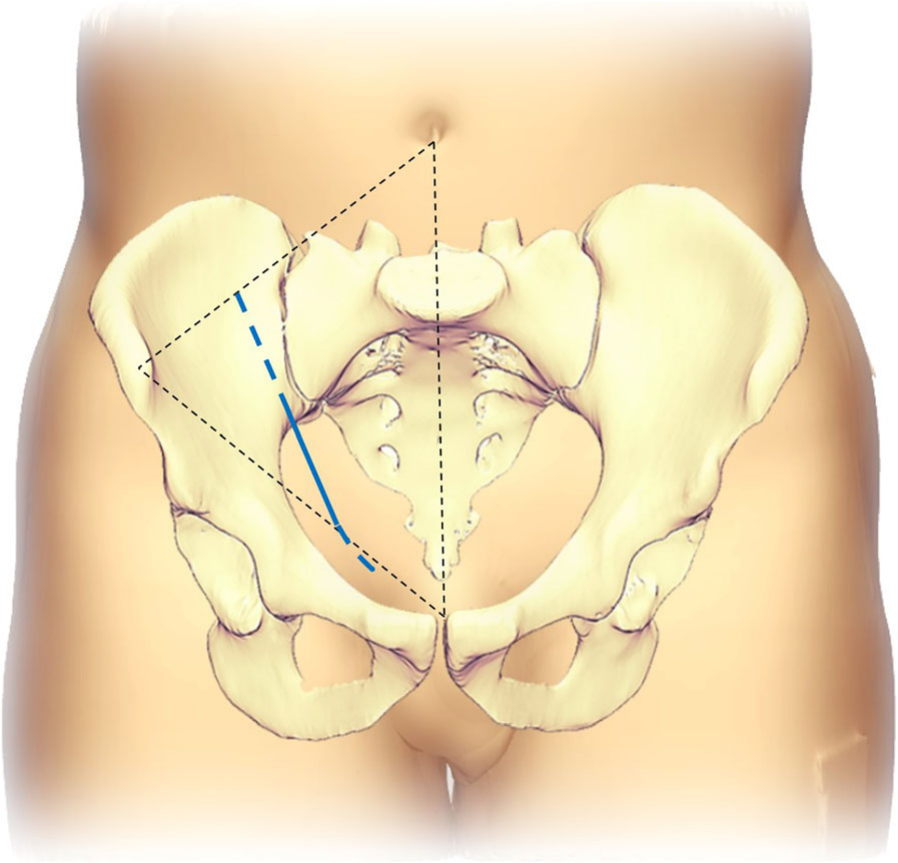
Diagram showing the incision of the limited approach, the inferior half of the pararectus approach was used (solid blue line), and the incision could be extended bilaterally to be a standard pararectus approach if needed

**Fig. 2:**
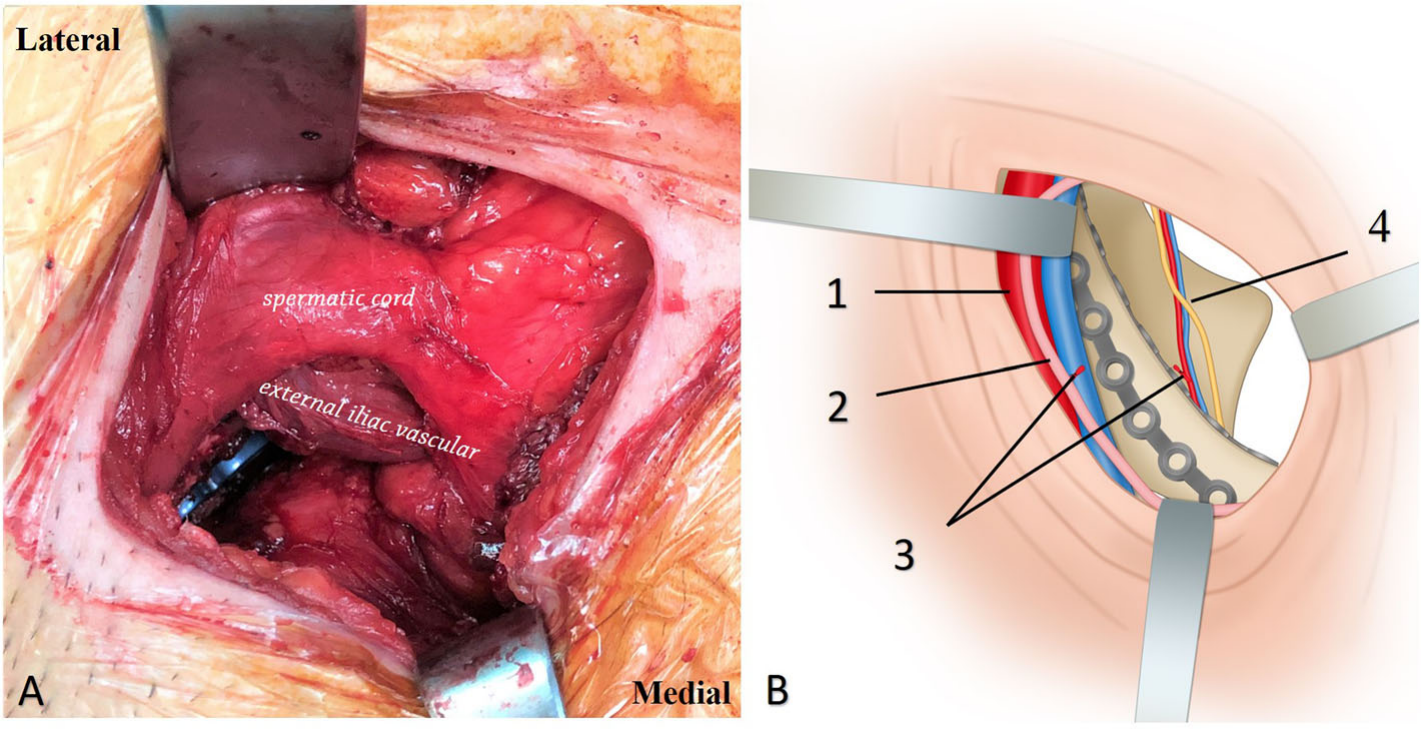
**a** Medial window of the pararectus approach was accessed to enter the space between the external iliac vascular, inguinal ligament and rectus abdominis. **b** Diagram showing the visualization of the limited approach, 1 external iliac vascular bundle, 2 spermatic cord/ round ligament, 3 anastomosis between the inferior epigastric vascular/ external iliac vessels and obturator vessels (corona mortis, ligated), 4 obturator nerve and vessels

The patient was positioned supine on a flat radiolucent operative table allowing intraoperative fluoroscopic visualization. The ipsilateral limb was free to stretch with the hip joint flexed to relax the iliopsoas and external iliac/femoral neurovascular bundle. Chief surgeon was positioned on the contralateral side of the injured acetabulum. The skin incision was made with the inferior half of the pararectus approach [3] (Fig. 1). A mini-incision was made at the lateral edge of the rectus abdominis muscle, just medial to the external iliac neurovascular bundle and above the inguinal ligament. The approach was located in the triangle area between the external iliac vascular, inguinal ligament and rectus abdominis muscle. The sheath of rectus abdominis was partially dissected, while the inferior epigastric vascular bundle and spermatic cord/uterine round ligament were detached and drawn laterally with the external iliac neurovascular bundle (Fig. 2a). The fascia transversalis was separated bluntly to enter the extraperitoneal space. A S-hook was used to pull the peritoneum medially to protect the bladder and intestines. Anastomosis (corona mortis) between the inferior epigastric vascular/ external iliac vessels and obturator vessels was identified and ligated on the surface of the suprapubic root. The periosteum was dissected from the anterior pelvic brim to safely separate most of the quadrilateral plate and anterior wall of the acetabulum. The anterior aspect of the sacroiliac joint could be touched proximally. The obturator neurovascular bundle was carefully pulled downward together with the periosteum to avoid nerve injury (Fig. 2b).

**Fig. 3:**
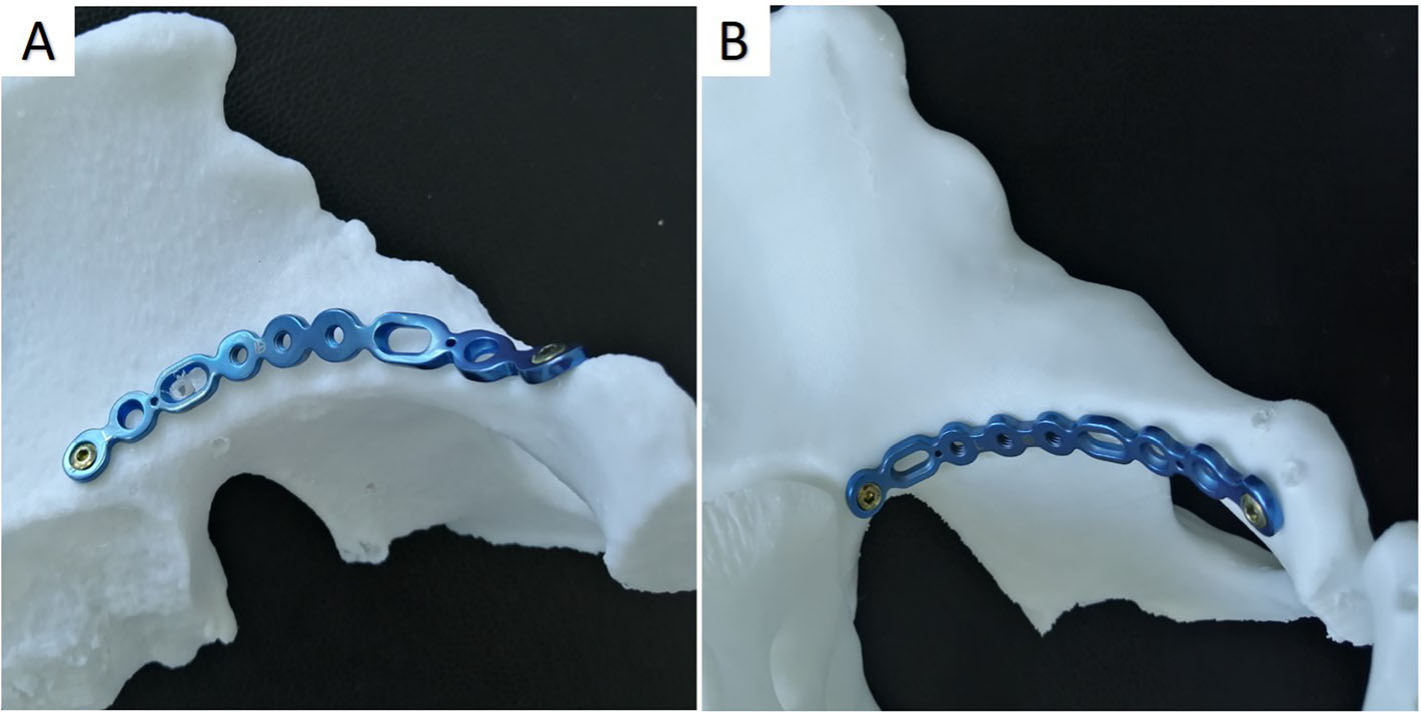
The new anatomical suprapectineal (**a**) and infrapectineal (**b**) plates have good congruity with the irregular acetabulum surface

Longitudinal and lateral traction facilitated reduction of displaced acetabular fractures. Reduction tools such as clamps and rods were used to reduce the displaced lower anterior column and quadrilateral plate fracture under direct vision. An anatomical plate was placed inferior the pelvic brim for firm fixation of the quadrilateral plate, and an anatomical suprapectineal plate was used for fixation of the anterior column and wall (Fig. 3).

### Imaging review

All patients received anteroposterior (AP) and Judet oblique views as well as CT scans pre- and post-operative. Standard procedures were used for multi-plane and three-dimensional (3D) reconstruction of CT images. A standardized procedure [18] was used to evaluate the residual step and gap displacement only in the weight-bearing dome (the upper 1 cm of the acetabulum) on postoperative CT scans. The greatest residual displacement in any of the axial, sagittal, or coronal plane views was measured and recorded. A circular template was drawn to fit along the acetabular articular surface and the gap displacement was measured along its perimeter. The step was obtained by the difference between the distance from the maximum displacement point of the fragment to the centre of the circle and the radius. An experienced radiologist (JL) and a trauma fellow (YLW) who were unaware of the patient’s surgical and clinical outcomes graded the reduction quality on postoperative X-ray images independently according to the Matta criteria [19]: anatomical (0–1 mm), imperfect (2–3 mm), or poor (> 3 mm). Imaging review was accomplished in the Picture Archiving and Communication Systems (PACS) and standard tools were used for the measurements.

### Postoperative management and follow-up

Functional muscle contraction training begins at the next day after surgery, and hip rehabilitation training progressively transitions from passive exercise to active exercise. Touchdown weight bearing was permitted the next day and continued for 6 weeks. Further partial weight bearing was permitted with progression to full weight-bearing. Patients were regularly followed up at an interval of 1 month, 3 months, 6 months and 1 year and then yearly if required. The modified Merle d’Aubigné and Postel score [20] was used to estimate the follow up functional outcomes. The sum of the individual scores (pain, gait, and range of motion of the hip) was classified as excellent (18 points), very good (17 points), good (15 or 16 points), fair (13 or 14 points), or poor (< 13 points).

## Results

The mean operative time was 90.9 ± 28.7 min (range: 45–150) and the mean blood loss was 334.1 ± 177.3 ml (range: 100–750). The mean residual gap and step displacement of the fractures was 2.9 ± 1.0 mm and 0.7 ± 0.6 mm, respectively. According to the Matta score, the quality of surgical reduction was graded as “anatomical” in 10 (58.8%) cases, “imperfect” in 6 (35.3%) cases, and “poor” in 1 (5.9%) case. All patients were regularly followed up for a period of at least 1 year, with the average 15 months (range: 12–27). One case of peritoneal injury occurred at the junction of the vas deferens and the peritoneum, and was repaired intraoperatively. Wounds healed within 2 weeks and there were no complications like infection and fat liquefaction. Functional healing of the fracture was achieved in all patients, with an average time of 12 weeks (range: 8–15), and no secondary displacement or implant failure was observed. Based on the Merle d’Aubigne and Postel score system [20], the functional outcomes were excellent in 9(52.9%) cases, good in 6(35.3%) cases, fair in 2(11.8%) cases (Table 2).

**Table 2:** Postoperative CT measurements, radiological and functional results (means ± standard deviation or number (percentage))

Parameter	value
CT measurements	
Mean maximal gap (mm)	2.9 ± 1.0
Mean maximal step (mm)	0.7 ± 0.6
Radiological outcome (Matta)	
Anatomical (< 1 mm)	10(58.8%)
Imperfect (2–3 mm)	6(35.3%)
Poor (> 3 mm)	1(5.9%)
Merle D’Aubigne-Postel score	
Excellent (18)	9(52.9%)
Good (15–17)	6(35.3%)
Fair (13–14)	2(11.8%)
Poor (< 13)	non

## Discussion

**Fig. 4:**
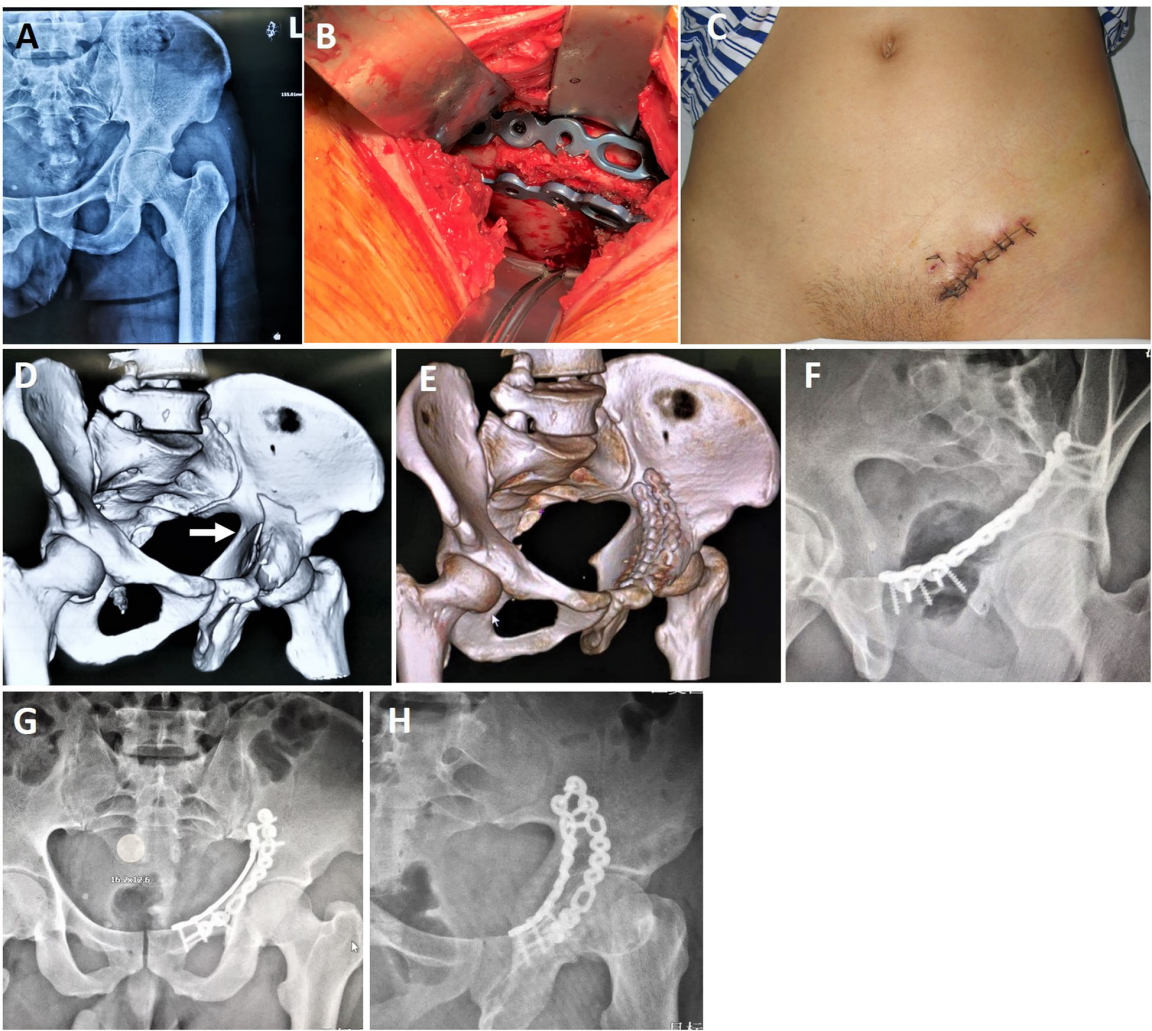
Case 6, a 45-year-old patient who sustained a fracture of the anterior column with medial subluxation. **a** Preoperative anteroposterior radiograph, **b** three hooks were used to exposed the anterior wall, pelvic brim and quadrilateral plate, **c** photograph showing the suture wound after operation, **d** preoperative 3D CT reconstructions, **e** postoperative 3D CT reconstruction showing the subluxation of the quadrilateral plate (white arrow), **f-h** postoperative anteroposterior and Judet oblique views

Extensive surgical approach for acetabular fractures is often accompanied by the possibility of perioperative complications such as infection, blood loss and neurovascular damage [3, 21]. Although closed reduction and minimally invasive surgery decrease surgical complications, but it is difficult to achieve anatomical reduction of articular surface [10]. The limited pararectus approach we used did not dissect the spermatic cord (or round ligament) and extra-iliac vascular nerve bundle, thereby reducing the risk of iatrogenic neurovascular injuries and complications. And it helped to achieve good reduction quality and functional results. Since it is located in the lateral margin of the rectus abdominis, the exposure of the anterior wall and quadrilateral plate will not be blocked by the rectus abdominis as happened in the modified Stoppa approach [22, 23]. We believe that direct and adequate exposure of fractures is instrumental in achieving anatomical reduction. On the other hand, owing to the limitation of minimally invasive incision, it is difficult and time-consuming to remodel the plate intraoperatively. The anatomical plate in this study is designed according to the physiological anatomical morphology, which makes it has good congruity to the irregular pelvis and has the function of auxiliary reduction (Fig. 4) [17].

The average operative time and blood loss were less than that of extensive surgical approach. Keel reported that the average operative time and the mean blood loss of the pararectus approach were 197 min and 1700 ml, respectively [3]. Using the modified Stoppa approach described by Sagi the operation time and blood loss were 263 min and 750 ml, respectively [16]. While by using an oblique mini-incision, Farouk [10] reported an average blood loss of 110 ml and an average operating time of 95 min. In our case series, the mean blood loss of 334.1 ml is more than Farouk `s, but significantly less than that of the established procedures published previously [1–3, 5]. Moreover, average operative time of 90.9 min in our sample, which is comparable to Farouk `s study. The wound of the limited pararectus approach is very small which may be the main reason for lower blood loss.

We consider that standardized image reading ensures rigorous evaluation of surgical reduction. On postoperative CT scans, the mean residual gap and step displacement was 2.9 mm and 0.7 mm, respectively. The anatomical and imperfect reduction rate was 94.1%. The functional outcome at the last follow-up were graded as good and excellent in 88.2% patients. The clinical result is comparable to the previous research [1, 3, 15, 16, 24]. Good radiological and clinical results are derived from the adequate visualization of fracture sites and valid fixation of the anatomical plates.

Traditionally, the suprapectineal and infrapectineal plates which are used to fix the anterior column and quadrilateral plate are usually inserted using the standard ilioinguinal and modified Stoppa approaches. The good congruity of the plate is obtained by reshaping the reconstruction plate intraoperatively. Owing to the limitation of the minimal incision in our study, the bone surface was not exposed fully, which made it difficult and time-consuming to reshape plate during operation. In recent years, some new anatomic plates instead of conventional reconstruction plate have been used for buttress and span fixation of the quadrilateral plate and medial surface of the posterior column [17, 25]. The anatomic plates have good biomechanical properties and congruity with the irregular acetabulum surface, which make the fixation more valid and easier. Moreover, in this study, we observed that the displacement between the fragment and the intact part was further reduced as the lag screw was inserted into the plate. Nevertheless, the relatively simple type of fractures in our cases facilitates the reduction, fixation and clinical outcome too. We consider that strict selection of patients is the key to ensure good surgical results, especially when using minimally invasive approaches.

The relative minimally invasive pararectus and modified Stoppa approaches are also accompanied with incidence of surgery-related complications ranging from 10.7 to 45.4% [13, 26]. It is easy to understand that extensive dissection of the neurovascular bundle and inguinal canal and intraoperative overstretching are supposed to be the main causes of surgical complications. The limited pararectus approach passes through the soft tissue space between the rectus abdominis and the neurovascular bundles to obtain closer access to anterior wall, anterior column and quadrilateral plate of the acetabulum. In this area, the extraperitoneal reflexed spermatic cord is the major obstacle of surgical exposure, pulling outward may be the cause of peritoneal rupture which occurred in one of our case. However, Both the wound and fracture were healed rapidly during follow-up, and no wound infection, fat liquefaction, ectopic ossification, or secondary displacement was recorded.

Closed reduction of displaced acetabular fractures may be minimally invasive, but finger palpation blindly for fracture manipulation and assessment of reduction may be inaccurate and may result in miss diagnosis of rotational displacement and more radiation exposure of medical staff [10]. However, in this study, the mini-pararectus approach might be a good choice for certain types of acetabular fractures involving low anterior column and quadrilateral plate, including anterior wall, anterior column with quadrilateral plate, simple transverse and anterior column with posterior hemitransverse fractures without iliac fragment. It can provide direct view through the soft tissue space, which decreases the risk of neurovascular injury and is beneficial to achieve anatomical reduction.

There are several limitations to this study. First, it is required to extend the minimal incision to the standard pararectus approach for the treatment of high anterior column fractures involving iliac wing and dome fragments in the elderly, which may be the main disadvantage of this approach. Besides, the minimal approach might not be suitable for delayed cases, patients with obesity or lower abdominal surgery history. Besides, we think the current problems of the two plates mainly include: (1) the length and model are invariable, so it is difficult to completely fix a long pelvic brim fracture; (2) the plasticity is not as good as that of stainless steel reconstruction plate, and excessive shaping will reduce the strength of the plate. This is a preliminary study on the use of the limited pararectus approach for the treatment of acetabular fractures. A prospective comparative study involving more cases needs to be further carried out, and long-term follow-up of patients is needed to be performed in the future.

## Conclusions

Based on the results of this study, the limited approach has advantages of less trauma, direct exposure to the anterior column and quadrilateral plate. This limited approach eliminates the need for direct dissection of important neurovascular bundle, avoids the obstruction of rectus abdominis, which makes it safe and easy. Intraoperative, this limited incision can provide sufficient view which improves the operation efficiency. The new anatomical plates can fit with the surface of acetabulum, which saves the time of remodeling plates during operation and facilitate fracture reduction. The combination approach can be a good choice for limited surgery of displaced anterior acetabular fractures especially involving the quadrilateral plate.

## Supplementary Information

**Additional file 1.**

## Data Availability

The datasets generated and/or analyzed during the current study are available from the corresponding author by reasonable request.

## Abbreviations

CT: Computerized tomography
SD: Standard deviation
ORIF: Open reduction and internal fixation
AP: Anteroposterior
3D: Three-dimensional
