# Supplementary material for: Internal fixation of anterior acetabular fractures with a limited pararectus approach and the anatomical plates: preliminary results
Source: BMC Musculoskelet Disord. 2021 Feb 18;22:203. doi: 10.1186/s12891-021-04034-w (PMC7891165; doi:10.1186/s12891-021-04034-w)
Supplement: Supplementary file 1 — Additional file 1. [file 12891_2021_4034_MOESM1_ESM.docx]

**Introduction to the supra-/infra- pectineal plates**

The plates are made of pure titanium (TA3) and is fitted to the pelvic brim. The supra-pectineal plate is a 2.8mm thick 9-hole plate (**Fig.1A**). Holes 1, 2, 8 and 9 are 3.5mm ordinary round holes, and 3.5mm screws can be placed. Holes 3 and 7 are oval sliding holes, and screw insertion point can be adjusted intraoperatively to place 3.5mm infra-acetabular screw and posterior column screw, respectively. Holes 4~6 are small round holes, corresponding to the joint surface area. In order to avoid the screw penetrating into the joint, no screw should be placed. The infra-pectineal plate is a 2.8mm thick 8-hole arc plate (**Fig.1B**). Holes 1~3, 7 and 9 are 3.5mm ordinary round holes that allows 3.5mm screws to be inserted. Holes 4 and 8 are oval sliding holes which allows 3.5mm screws to be inserted, or used as the joint holes of pubo-ischiatic plate and ilio-ischiatic plate respectively. Holes 5 and 6 are small round holes, corresponding to the joint surface, and screws are not inserted.

Therefore, the supra-/infra- pectineal plates are suitable for acetabular anterior column and quadrilateral plate fractures, and can be easily inserted with the pelvic rim exposed (**Fig.1C**).


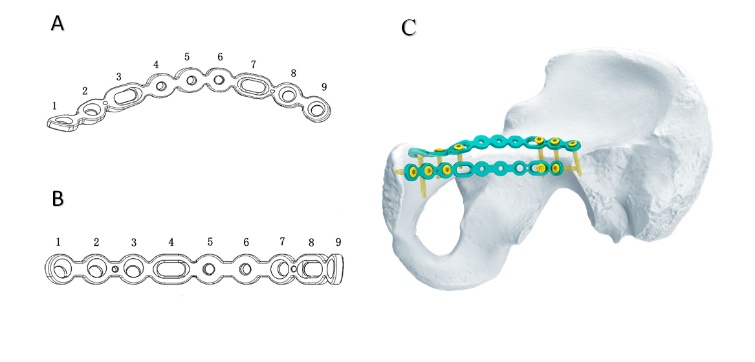


**Fig. 1** Schematic diagram of the two kinds of anatomical plates. **A** The supra-pectineal plate is a 9-hole plate fitted to the bone surface of the anterior column. B The supra-pectineal plate is a 8-hole placed below the iliopectineal line to resist medial displacement of the quadrilateral plate. **C** The plates are fit to the irregular pelvic brim.
